# Antimicrobial resistance and over the counter use of drugs in Nepal

**DOI:** 10.7189/jogh.10.010360

**Published:** 2020-06

**Authors:** Sunil Pokharel, Bipin Adhikari

**Affiliations:** 1 Centre for Tropical Medicine and Global Health, Nuffield Department of Medicine, University of Oxford, Oxford, UK; 2 Nepal Community Health and Development Centre, Balaju, Kathmandu, Nepal

Antimicrobial resistance (AMR) is an emerging global threat. Antimicrobial resistant infections cause 700 000 deaths every year globally and is estimated to account for 10 million deaths each year by 2050 if no actions taken [1]. World Health Organization has warned that the current trend in the emergence and spread of AMR could lead to ‘post-antibiotic era’ catastrophic consequences [2]. In Asia alone, more than 4 million deaths are projected to be due to AMR by 2050 [3].

## BURDEN OF AMR IN SOUTH ASIA

The risk and burden of AMR in Asia are disproportionately higher than any other continents because of high population growth triggering antimicrobial demand together with the unregulated antimicrobial use in both humans and animals, and the wide circulation of counterfeit and sub-standard medicines [4]. Asia is the most populous continent of the planet earth with the population of 4.5 billion and constitutes 60% of the world’s population with China and India together constituting more than two-third of the Asia’s population [5]. Together with the population density, current transition in economy, migration, high burden of infectious diseases and tropical diseases and the consequent demands and use of antimicrobials has made Asia a hub for the antimicrobial resistance [6]. The emerging resistance of antimicrobials in South Asia is intertwined with epidemiological, social, cultural and political characteristics of the nations. In this article, we will discuss the social, cultural and regulatory factors contributing to AMR and potential solutions by focusing on the context of Nepal.

## AMR IN THE CONTEXT OF NEPAL

In Nepal, the doctor patient ratio is still below (0.17/1000 population) the WHO recommendation (2.3/1000 population) [7]. The inequality in the distribution of health facilities and trained health human resources between urban and rural areas is alarmingly high. A huge proportion of population is deprived of the optimal health facilities creating a conducive environment for informal health care services to grow. The financial incentives to the dispensers together with poorly regulated medicine dispending practice and consumers’ concern about the cost of consultation and investigations in hospital visits largely drive the over the counter (OTC)/non-prescription use of antimicrobials. The widespread myths that ‘antibiotic’ treat all forms of infections and are effective against viral infections undermine the role of a medical consultation [8]. Such practices of prescribing antimicrobials have been in annual rise.

In Nepal, apart from public health facilities and private hospitals/clinics, there are huge number of formal/informal pharmacies similar to grocery shops. Drug Act 1978, and subsequent regulator frameworks have clearly mentioned antibiotics as the drug requiring prescription for dispensing. Nepal Pharmacy Council has outlined the guideline for the dispensing of prescribed drugs by a qualified and registered pharmacists [9]. However, the practice in drug-shops ubiquitous in Nepal is largely unregulated and drug dispensers have poor compliance to good pharmacy practice [10]. Hospital pharmacies located around the premises of government and private hospitals are under-equipped, under-resourced and are not readily accessible to a large population. Community pharmacies (often non-pharmacist run registered/unregistered ‘drug shops’) both in urban and rural Nepal generally practice in irrational dispensing of the antibiotics. These unregulated ‘drug shops’ sell wide range of prescription only medicines which commonly include antimicrobials and often provide in higher doses exceeding the therapeutic limit and sometimes in sub-therapeutic doses [11]. In addition, in most of the cases, antimicrobials are empirically dispensed without establishing a diagnosis.

OTC use of antimicrobial occurs when patients use medicines without a prescription from medical practitioner and without a medical supervision. OTC medications for common infections such as respiratory tract infections, urinary tract infections and typhoid fever by minimally qualified and unqualified allied health professionals is a common practice in developing countries where more than 50% antimicrobials are used without a medical prescription, most commonly purchased from pharmacies or ‘drug shops’ [12]. A recent systematic review reported a high prevalence of self-medication with antimicrobials, most commonly amoxicillin, macrolides, fluoroquinolones, cephalosporins and metronidazole in Southeast Asia [13]. Antimicrobial use exerts antimicrobial selection pressure and contributes to the development of AMR [14,15]. This is more pronounced with OTC associated with inappropriate selection of antimicrobial drugs, their dose and duration of treatment [14]. Primary care facilities including the symptomatic treatment of infections were found to be the prominent reasons for high odds (range 1.4-3.7) of developing AMR [12,14].

Healthcare expenditure as a share of national GDP in Nepal has largely increased over the past decade mostly driven by out of pocket (OOP) expenditure (OOP contributed more than 60% of health care expenditure) [16]. Government expenditure on health has remained low, 11% population had health care expenditure of more than 10% of total expenses and 1.7% of total population was pushed below poverty line in 2016 due to health care expenses [16]. Public health delivery system is ineffective even in urban areas due to poor governance, lack of skilled human resources and quality laboratory services. Private health facilities are expensive (high cost associated with diagnosis, treatment, travel and opportunity cost) and out of reach to a large population. Government of Nepal has recently implemented social health insurance system which is challenged by low acceptability and coverage (5% in the implemented districts) and is a long way from significantly contributing to universal health coverage [17]. The huge OOP health expenditure has rendered relatively cheaper OTC a first option in health care seeking and hospital consultation a last resort.

Apart from apparent financial shortcuts of visiting pharmacies, in Nepal and in other South Asian countries, both formal and informal Ayurvedic and traditional practitioners are attended by large proportion of the population [18]. Unregulated Ayurvedic and traditional practitioners often empirically use antimicrobials either in the form of certified allopathic product or uncertified plant extracts with antimicrobial property to treat infections. Such a practice, in fact is distracting the early health seeking behavior. Augmented by the socio-demographic barriers (distance, direct and indirect costs associated with attending hospital), traditional beliefs and practices, patient may easily fall into a cycle of poly visits to traditional healers and medical dispensers thus propitiating the development of AMR.

**Figure:**
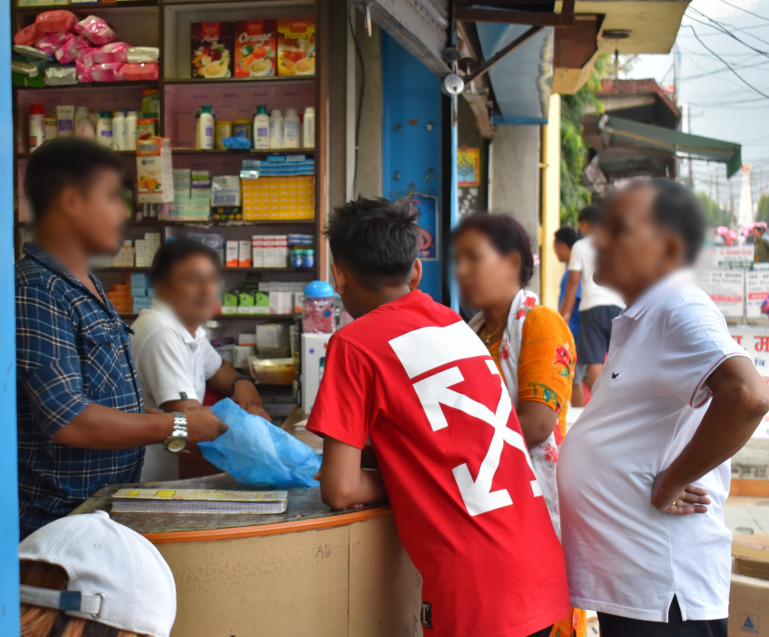
Photo: From the collection of Dr Achyut Adhikari (used with permission).

## WAY FORWARD

Legislation in Nepal mandates prescription for dispensing of an antimicrobial drug by a registered pharmacist. Strict enforcement of regulation, particularly in dispensing of the antibiotics by the pharmacies, together with development and adherence to national standard treatment protocol, is urgently required. Universal health care with increased access to public health facilities and health insurance for financial protection against catastrophic private health care expenditure is critical. Nevertheless, these interventions alone are insufficient to stop OTC dispensing of antimicrobials in the socio-economic context of Nepal and will be futile without addressing the social, cultural and behavioral factors driving the OTC antimicrobial use. Research to understand the trends in antimicrobial use and factors driving the OTC antimicrobial use, and follow-up address to the knowledge gaps through policy interventions is urgently required.
